# Pubertal Body Mass Index Change Is Associated With Adult Coronary Atherosclerosis and Acute Coronary Events in Men

**DOI:** 10.1161/ATVBAHA.121.316265

**Published:** 2021-06-17

**Authors:** Jenny M. Kindblom, Maria Bygdell, Ola Hjelmgren, Jari Martikainen, Annika Rosengren, Göran Bergström, Claes Ohlsson

**Affiliations:** 1 Department of Internal Medicine and Clinical Nutrition, Institute of Medicine, Centre for Bone and Arthritis Research, the Sahlgrenska Academy at University of Gothenburg, Sweden (J.M.K., M.B., C.O.).; 2 Region Västra Götaland, Sahlgrenska University Hospital, Pediatric Clinical Research Center, Gothenburg, Sweden (J.M.K.).; 3 Department of Molecular and Clinical Medicine, Institute of Medicine, Sahlgrenska Academy, University of Gothenburg, Sweden (O.H., A.R., G.B.).; 4 Region Västra Götaland, Sahlgrenska University Hospital, Department of Clinical Physiology, Gothenburg, Sweden (O.H., G.B.).; 5 Bioinformatics Core Facility, the Sahlgrenska Academy at the University of Gothenburg, Sweden (J.M.).; 6 Region Västra Götaland, Sahlgrenska University Hospital, Department of Medicine/Östra, Gothenburg, Sweden (A.R.).; 7 Region Västra Götaland, Sahlgrenska University Hospital, Department of Drug Treatment, Gothenburg, Sweden (C.O.).

**Keywords:** atherosclerosis, acute coronary events, body mass index, overweight, puberty, risk factors

## Abstract

**Objective:**

The aim with the present study was to evaluate the association between pubertal body mass index (BMI) change and adult coronary artery calcification (CAC) score and risk of acute coronary events.

**Approach and Results:**

We included 37 672 men from the BMI Epidemiology Study and calculated their pubertal BMI change (BMI at 20 years−BMI at 8 years). Coronary artery computed tomography analysis of CAC score, midlife BMI, and major risk factors for coronary heart disease were available for a sub-cohort through linkage with the SCAPIS (Swedish Cardio Pulmonary Bioimage Study) cohort (n=922). Information on first acute coronary events was retrieved from Swedish national registers (n=37 672, events n=1873). Pubertal BMI change (odds ratio per SD increase, 1.32 [1.14–1.52]), but not childhood BMI, was associated with middle age CAC score ≥1. This association for pubertal BMI change was maintained after adjustment for midlife BMI at CAC analysis and in a model including major cardiovascular risk factors. Individuals who became overweight during puberty (hazard ratio, 2.11 [1.79–2.49]), but not those overweight at 8 years who normalized their weight during puberty, had substantially increased risk of acute coronary events compared with men who were never overweight. Among subjects with an acute coronary event, individuals with pubertal onset overweight were at increased risk of death due to the event.

**Conclusions:**

Pubertal BMI change is an independent predictor of CAC score and risk of acute coronary events in adult men. Excessive BMI increase during puberty may initiate the coronary atherosclerotic process, thereby increasing the risk and severity of adult acute coronary events.

## Abstract 2

Supplemental Digital Content is available in the text.

**Highlights**

- The pubertal body mass index (BMI) change (BMI at 20 years minus BMI at 8 years of age), but not BMI at 8 years of age, is associated with a coronary artery calcification score ≥1.
- The association between the pubertal BMI change and coronary artery calcification score is maintained after adjustment for midlife BMI. The major cardiovascular risk factors cholesterol level, systolic blood pressure, and smoking are not mediators in this association.
- Individuals who became overweight during puberty and who were overweight throughout childhood and puberty, but not those overweight at 8 years who normalized their weight during puberty, had substantially increased risk of acute coronary events compared with men who were not overweight at childhood or after puberty.
- Among subjects with an acute coronary event, individuals with pubertal onset overweight were at increased risk of death due to the event.

**See accompanying editorial on page 1617**

Coronary heart disease (CHD) is the leading cause of death globally^1^ and with obesity as an important risk factor behind CHD, the recent obesity epidemic among children as well as adults is a major health concern.^2–4^ Some epidemiological studies have described an association between body mass index (BMI) in childhood and coronary events in adulthood.^5,6^ Two recent publications using the Mendelian randomization approach in the large UK Biobank cohort confirm the association between self-reported size in childhood and adult CHD,^7^ and BMI during childhood and adult asthma,^8^ but further demonstrate that the associations are mainly mediated through high BMI in midlife.^7,8^ Furthermore, a high BMI in late adolescence has been reported to associate with increased risk of CHD.^9–11^ Taken together, there is observational evidence of an association between a high BMI both in childhood and adolescence and adult CHD, but the relative contribution of childhood BMI, pubertal BMI change, and midlife BMI, and the mechanisms behind this association, are not clear. We have previously demonstrated that the correlation between childhood BMI and pubertal BMI change is marginal, indicating that these 2 developmental parameters, reflecting childhood and puberty, have the potential to contribute nonoverlapping information as risk markers for adult disease.^12^ This is further supported by our previous findings that pubertal BMI change, but not childhood BMI, predicts cardiovascular mortality,^12^ stroke,^13^ heart failure,^14^ and asthma.^15^ However, the associations between pubertal BMI change and coronary artery atherosclerosis and the risk of adult acute coronary events have not been evaluated. Furthermore, it is not known whether pubertal BMI change may affect the atherosclerotic disease process independently of midlife BMI.

As part of the population-based BEST (BMI Epidemiology Study) in Gothenburg, Sweden, information on childhood BMI and young adult BMI shortly after puberty has been collected for men born between 1945 and 1961. The recruitment base of this cohort overlaps with the Gothenburg parts of the SCAPIS (Swedish Cardio Pulmonary Bioimage Study), with a thorough cardiovascular characterization including coronary artery calcification (CAC) score using computed tomography.^16^ This overlap between the cohorts gives a unique opportunity to study the association between BMI during development and both adult coronary atherosclerosis and risk of adult acute coronary events in individuals from the BEST Gothenburg cohort. We have previously demonstrated that pubertal BMI change, but not childhood BMI, is independently associated with cardiovascular mortality.^12^ In the present study, we evaluated our hypothesis that pubertal BMI change is, independently of midlife BMI, associated with midlife CAC score, reflective of the atherosclerotic process in the coronary arteries, and with incidence and severity of acute coronary events.

## Methods

The data that support the findings of this study are available from the corresponding author upon reasonable request and upon approval from the University of Gothenburg according to mandatory national law but are not publicly available due to privacy and ethical restrictions.

### Study Population

#### The BMI Epidemiology Study Gothenburg

The population-based BEST Gothenburg cohort was initiated with the overall aim to study the impact of BMI during childhood and puberty on adult diseases as previously described.^12–14^ We collected data on height and weight during development from school health care records and at young adult age from military conscription tests (see also Appendix and Table I in the Data Supplement). Because almost exclusively men underwent conscription, pubertal BMI change could only be estimated in males. The ethics committee of the University of Gothenburg, Sweden approved the BEST Gothenburg study.

#### The Linked BEST Gothenburg-SCAPIS Sub-Cohort

SCAPIS is a prospective observational study^16^ with data collection at 6 university hospitals in Sweden and a thorough cardiovascular characterization including computed tomography of the coronary arteries. For a more detailed description of the SCAPIS cohort, see Appendix in the Data Supplement. Written and oral consent was obtained from all SCAPIS subjects.

The BEST Gothenburg cohort shares its recruitment base with the SCAPIS pilot study and SCAPIS Gothenburg. Through linkage between the BEST Gothenburg and the SCAPIS cohorts using the individuals’ personal identity number, we obtained information on CAC score, midlife BMI, and risk factors for cardiovascular disease from SCAPIS linked to information on developmental BMI for a sub-cohort of the BEST Gothenburg cohort that overlaps with SCAPIS (n=922). The linked BEST Gothenburg—SCAPIS sub-cohort is representative of the population-based complete BEST Gothenburg cohort (See results in the Data Supplement and Table). The ethics committee of the University of Gothenburg, Sweden, approved the BEST Gothenburg—SCAPIS linkage study.

**Table.**
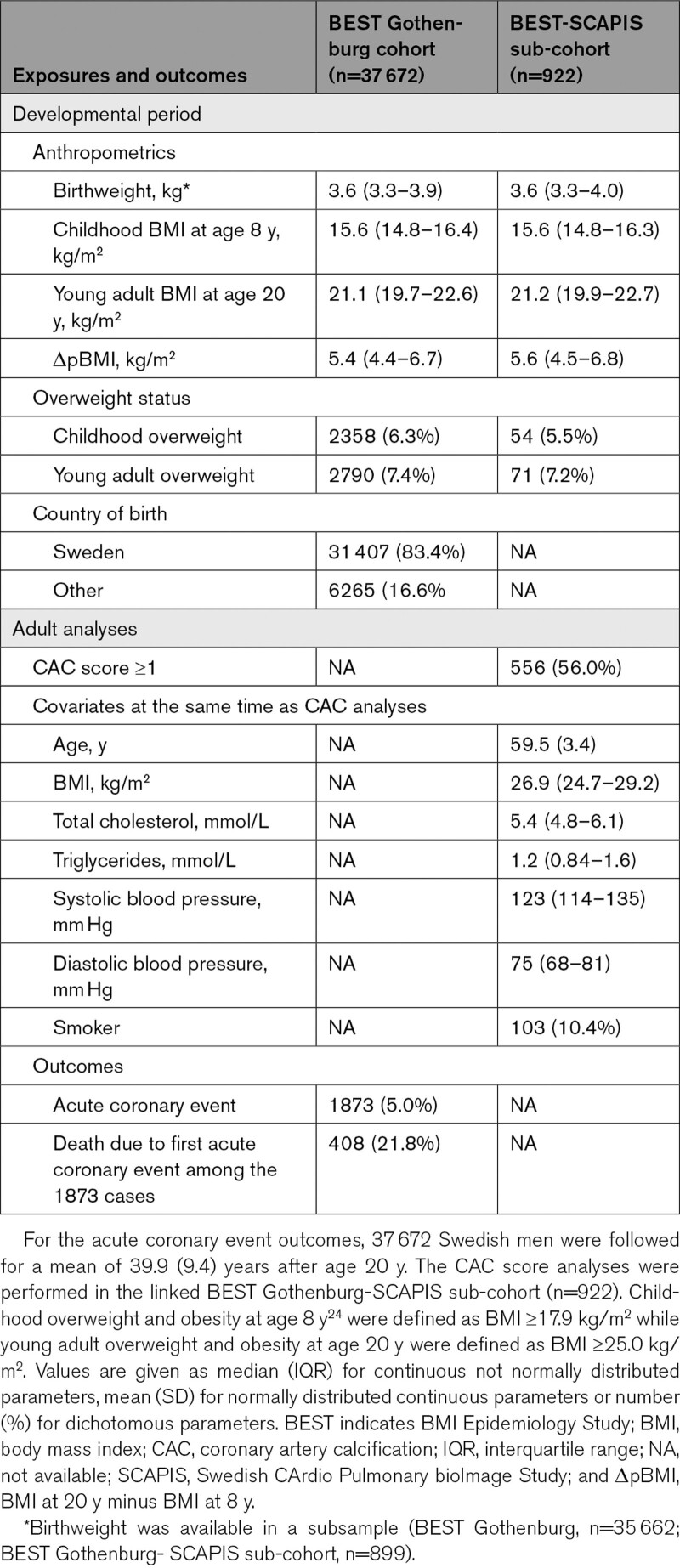
Cohort Description of the BMI Epidemiology Study Gothenburg Cohort and the BEST Gothenburg-SCAPIS Sub-Cohort

### Exposures From the BEST Gothenburg Study

Prepubertal childhood BMI at 8 years of age and young adult BMI at 20 years of age were calculated using all paired height and weight measurements in the period between 6.5 and 9.5 years of age for prepubertal childhood BMI, and in the period 17.5 to 22 years of age for young adult BMI as previously described^12^ (see Materials in the Data Supplement). Pubertal BMI change was defined as the difference between young adult BMI (at age 20) and childhood BMI (at age 8).

### CAC Score, Midlife BMI, and Cardiovascular Risk Factors From SCAPIS

#### Midlife CAC Score

**Figure 1.**
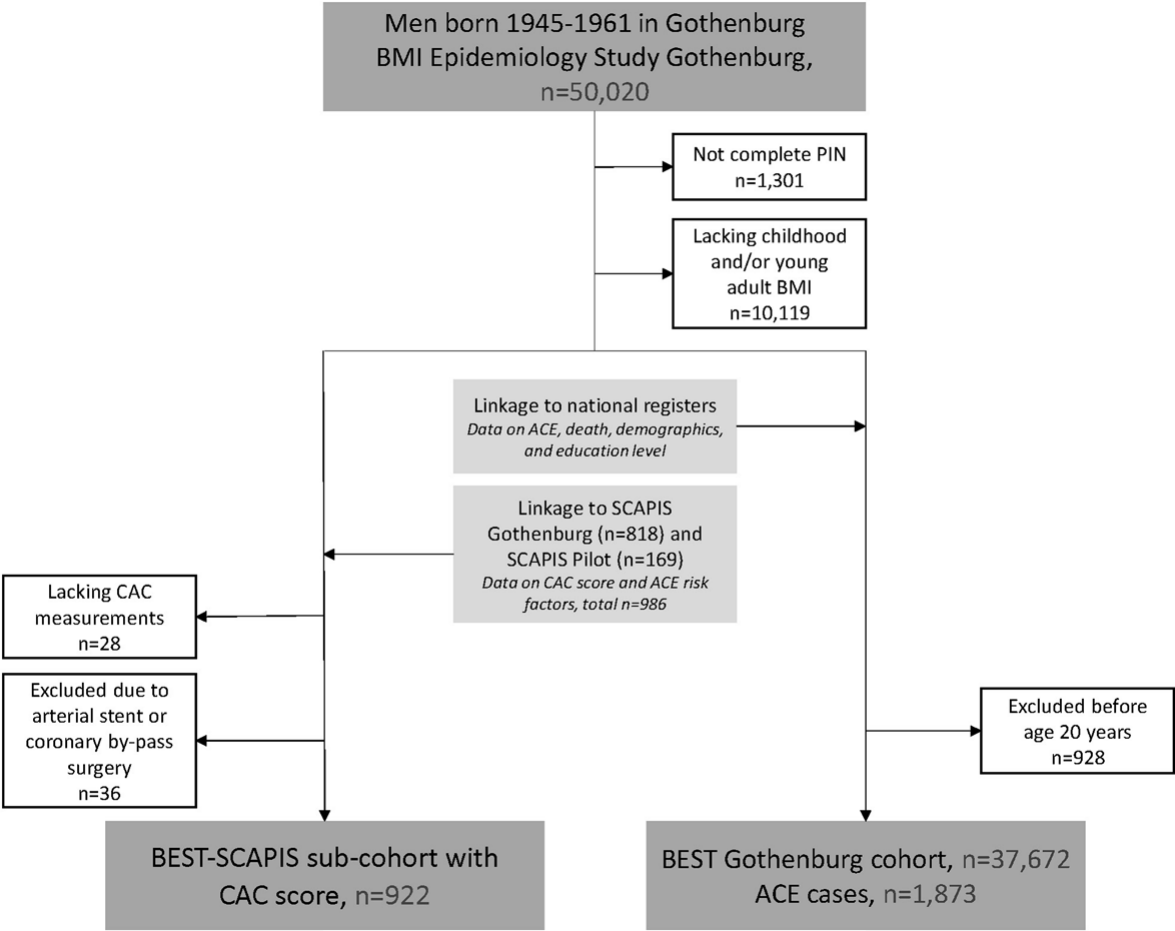
**Flow chart of included individuals.** ACE indicates acute coronary events; BEST, BMI Epidemiology Study; BMI, body mass index; CAC, coronary artery calcification; PIN, personal identity number; and SCAPIS, Swedish Cardio Pulmonary BioImage Study.

CAC was assessed using a state-of-the-art multi-slice computed tomography scanner (Siemens, Somatom Definition Flash, Siemens Medical Solution, Forchheim, Germany, see also Materials in the Data Supplement). The calcium content in each coronary artery was measured, summed, and quantified using the Agatston score.^17^ An Agatston score ≥1 was defined as having CAC. This level has previously been demonstrated to be significantly associated with increased CHD risk and to indicate atherosclerosis in the coronary arteries.^18^ CAC score was not measured in the presence of a cardiac stent or if there was a history of a previous by-pass surgery (Figure 1).

#### Midlife BMI

Body weight at physical examination in SCAPIS was measured on a balance scale with subjects dressed in light indoor clothing without shoes. Body height was measured to the nearest centimeter. Midlife BMI was calculated as weight in kilograms divided by height in meters squared.

#### Midlife Cardiovascular Risk Factors

Systolic and diastolic blood pressures were measured twice in each arm with an automatic device (Omron M10-IT, Omron Health care Co, Kyoto, Japan), and the mean of the measurements was used. Total cholesterol was measured in venous blood after an overnight fast (Cobas 8000 Roche Diagnostica Scandinavia AB). Smoking was self-reported in questionnaires as current smoker or past/never smoker.

### Outcome From National Registers for the BEST Gothenburg Cohort

The 37 672 men in the BEST Gothenburg cohort were followed from 20 years of age until an acute coronary event, or censoring due to death or migration, or until December 31, 2016, whichever came first. The outcomes, acute coronary event and death due to acute coronary event, were defined according to the *International Classification of Diseases* system (Table II in the Data Supplement). Death due to a coronary event was defined as death within 28 days after the event.

### Statistical Analyses

We used an age-adjusted logistic regression model to calculate odds ratios for a CAC score ≥1 (ie, adjusted for age at CAC score measurement). The model was further adjusted for midlife BMI at CAC score measurement and major CHD risk factors. Cox proportional hazards regressions were used to analyze the association between exposures (childhood BMI and pubertal BMI change) and acute coronary events. The Cox regression model was adjusted for country of birth and birth year in Figure 3, for birth year, country of birth, and birthweight in Table III in the Data Supplement, for birth year, country of birth, and education level in Table IV in the Data Supplement, for birth year in Table V in the Data Supplement and for country of birth and birth year in Table VI in the Data Supplement. A *P* value of <0.05 was interpreted as statistically significant. The assumption of proportional hazards was confirmed for all variables in the model including overweight groups. However, in the model containing pubertal BMI change as a continuous parameter, we detected violation of the assumption of proportional hazards and therefore, the follow-up in subsequent analyses was split on the median age at diagnosis (56.7 years) and the associations are presented separately for risk of early (≤56.7 years of age) and late (>56.7 years of age) acute coronary events. Birth weight, childhood BMI (at 8 years), and young adult BMI (at 20 years) were not normally distributed and have been log-transformed in analyses where they are included as continuous variables. Kaplan-Meier survival plot of survival free of acute coronary events was performed with subjects categorized according to overweight status at 8 and 20 years of age and significance tested using a log-rank test between the groups with adjustment for multiple testing (3 comparisons). We used a cumulative incidence plot to assess competing risk. Missing values were not imputed.

**Figure 2.**
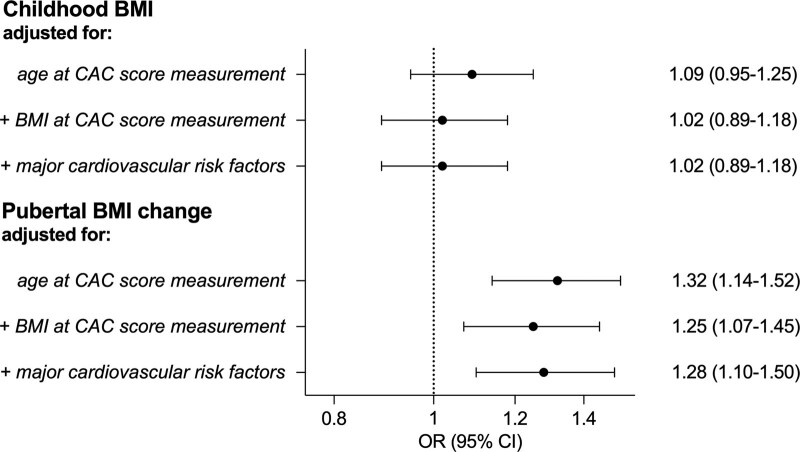
**Associations between pubertal body mass index (BMI) change and coronary artery calcification (CAC) score in the BEST-SCAPIS (BMI Epidemiology Study, Swedish Cardio Pulmonary BioImage Study) sub-cohort.** Odds ratios (ORs; 95% CI) per SD increase for childhood BMI (**upper**) and pubertal BMI change (**lower**) in relation to a CAC score ≥1 were calculated using logistic regression models (n=922). The different models were adjusted as indicated in the figure first in both the **upper** and **lower** part for age at CAC score measurement (n=922), in the middle analysis in both parts for age at CAC score analysis and midlife BMI at the time of the CAC score measurement (n=922), and in the **bottom** analysis in both parts for age at CAC score measurement, midlife BMI and the major risk factors for coronary heart disease; smoking (n=922), systolic blood pressure (n=922), and cholesterol levels (n=917) determined at the time of the CAC score measurement.

## Results

### Pubertal BMI Change Displays Modest Associations With Both Childhood BMI and Midlife BMI

We followed 37 672 Swedish men born 1945 to 1961 from the BEST Gothenburg cohort with data on both childhood BMI at 8 years of age and young adult BMI at 20 years of age (Figure 1). Through linkage with the SCAPIS study, to form the BEST-SCAPIS sub-cohort, we retrieved data on midlife CAC score, midlife BMI, and major risk factors for CHD measured at the time of CAC score measurement (mean age at CAC score measurement 59.5 years [3.4]; n=922; Table). Among the 37 672 in the complete BEST Gothenburg cohort, 1873 first acute coronary events (fatal and nonfatal) were diagnosed and retrieved from national registers during 1 504 242 person-years follow-up (mean follow-up, 39.9 [9.4] years) from age 20 years until December 31, 2016 (Table). The mean age at acute coronary event diagnosis was 55.9 [7.8] years. The correlation between childhood BMI and pubertal BMI change was marginal in the linked BEST—SCAPIS sub-cohort (Pearson’s *r*=0.04, n=922), similarly as previously reported for the complete BEST Gothenburg cohort.^12^ A modest correlation between pubertal BMI change and middle age BMI (Pearson’s *r*=0.37; variance explained [*r*^2^]=14%) was found in the linked BEST-SCAPIS sub-cohort. Thus, pubertal BMI change contributes information that is largely (86%) nonoverlapping with midlife BMI, demonstrating that pubertal BMI change may have the potential to be an independent risk marker for disease.

### Associations Between Pubertal BMI Change and CAC Score in the BEST-SCAPIS Sub-Cohort

The linked BEST -SCAPIS sub-cohort was used to evaluate the association between the developmental BMI parameters and CAC score, (n=922; Figure 1). The CAC score was dichotomized into no coronary calcification (CAC score, 0), or coronary calcification (CAC score, ≥1). As many as 556 (56.0%) of the men had a midlife CAC score ≥1. Childhood BMI was not associated with CAC score in any model evaluated (Figure 2). In contrast, an age-adjusted logistic regression model revealed that pubertal BMI change was significantly associated with CAC score (Figure 2), and adjustment for childhood BMI did not alter the observed direct association between pubertal BMI change and CAC score (odds ratio per SD increase, 1.31 [1.14–1.52]). Importantly, the significant association between pubertal BMI change and coronary calcification was maintained after adjustment for midlife BMI measured at the time of CAC score measurement (59.5 [3.4] years; Figure 2). We next evaluated the association between pubertal BMI change and 3 major cardiovascular risk factors that besides midlife BMI may mediate the association between pubertal BMI change and CAC score. The pubertal BMI change was modestly associated with cholesterol levels (SD change per SD increase in pubertal BMI change, −0.075 [95% CI, −0.14 to −0.01]) and systolic blood pressure (SD change per SD increase in pubertal BMI change, 0.080 [95% CI, 0.02–0.14]), while it was not significantly associated with smoking status (odds ratio per SD increase in pubertal BMI change, 1.06 [95% CI, 0.86–1.29]; all models adjusted for age at risk factor measurement). In addition, when these 3 cardiovascular risk factors were included as covariates, the strength of the association between pubertal BMI change and CAC score was not attenuated, suggesting that it is unlikely that the observed association is mediated to a substantial part via any of these 3 risk factors (Figure 2). We also evaluated the association between pubertal BMI change and CAC score ≥100 (odds ratio, 1.41 [95% CI, 1.16–1.72]; full model [CAC score 0, referent]).

### Associations Between Childhood and Young Adult Overweight Groups and the Risk of Adult Acute Coronary Events in the BEST Gothenburg Cohort

**Figure 3.**
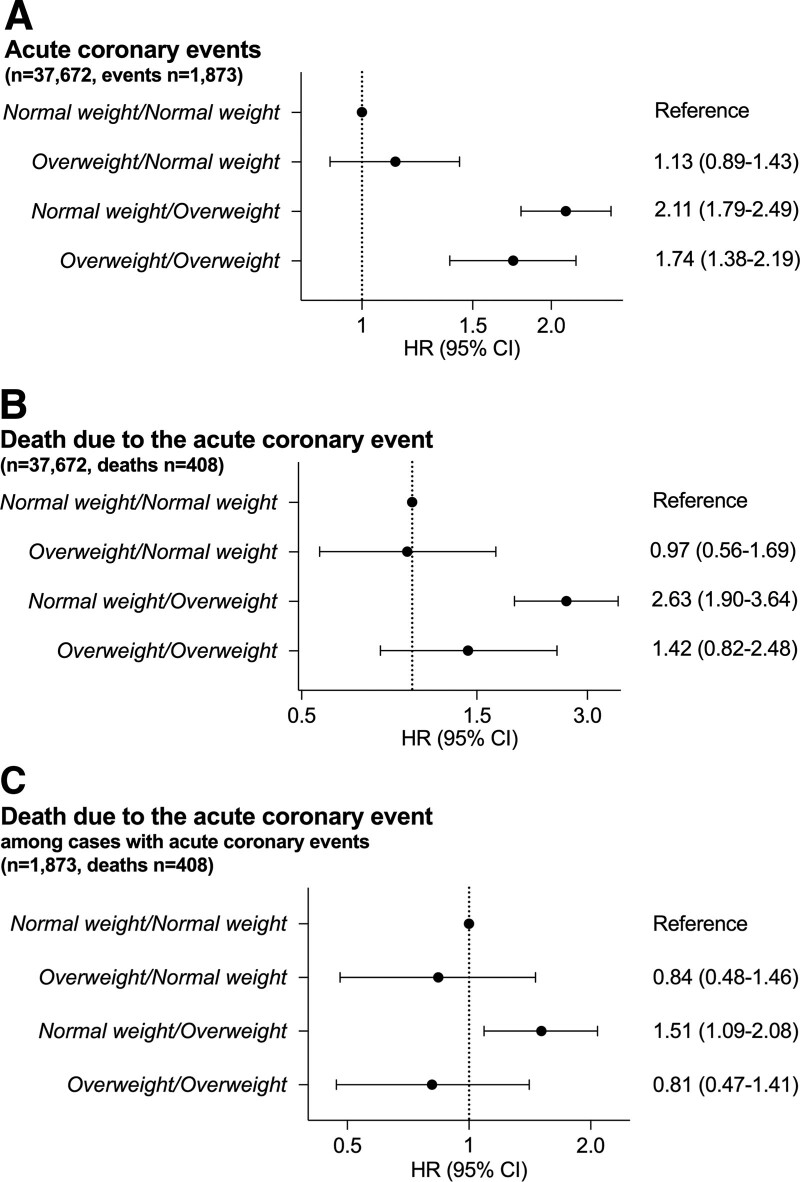
**Associations between childhood and young adult overweight groups and the risk of adult acute coronary events and death due to the event in the BEST (BMI Epidemiology Study) Gothenburg cohort. A**, Acute coronary events: hazard ratios (HRs) for the risk of an acute coronary event in the complete cohort (n=37 672) were calculated using Cox proportional hazards regression adjusted for birth year and country of birth. Normal weight/normal weight (=reference; normal weight at both 8 and 20 y of age) n=33 514 (event cases: 1573), overweight/normal weight (overweight at 8, normal weight at 20) n=1368 (70), normal weight/overweight (normal weight at 8, overweight at 20) n=1800 (156), overweight/overweight (overweight at both 8 and 20 y of age) n=990 (74). **B**, Death due to the acute coronary events: HRs for the risk of death due to the acute coronary event in the complete cohort (n=37 672) were calculated using Cox proportional hazards regression adjusted for birth year and country of birth. Normal weight/normal weight (=reference) n=33 514 (341 died), overweight/normal weight n=1368 (13 died), normal weight/overweight n=1800 (41 died), overweight/overweight n=990 (13 died). **C**, Death due to the acute coronary event among individuals with an acute coronary event: HRs for the risk of death due to the acute coronary event among individuals with an acute coronary event (n=1873) were calculated using Cox proportional hazards regression adjusted for birth year and country of birth. Normal weight/normal weight n=1573 (=reference; 341 died), overweight/normal weight n=70 (13 died), normal weight/overweight n=156 (41 died), overweight/overweight n=74 (13 died). Childhood overweight at 8 y of age^24^ was defined as body mass index (BMI) ≥17.9 kg/m^2^ while young adult overweight at 20 y of age was defined as BMI ≥25 kg/m^2^. All models have been adjusted for birth year and country of birth.

Using the complete BEST Gothenburg cohort linked with information from national Swedish registers (n=37 672), we evaluated the associations between overweight during childhood and young adulthood and the risk of adult acute coronary events. Individuals who became overweight during puberty, that is, with normal weight at age 8 years and overweight at age 20 years, had a substantially increased risk of an acute coronary event in adulthood compared with men who were never overweight (Figure 3A). The group that was overweight throughout childhood and adolescence also had an increased risk compared with the normal weight group. In contrast, the group with overweight at 8 years of age that normalized during puberty, that is, normal weight at age 20, did not have an increased risk of an adult acute coronary events compared with the group with normal weight throughout childhood and young adulthood (Figure 3A). In total, 408 of the 1873 men with an acute coronary event died within 28 days from the event. Interestingly, when we evaluated the association between developmental overweight status and death due to the acute coronary events, only the group that was normal weight at age 8 years and overweight at 20 years had significantly increased risk of death due to the event, compared with individuals with normal weight at both 8 and 20 years. No significantly increased risk was seen for the group with overweight at age 8 followed by normal weight at 20 years, or the group that was overweight at both 8 and 20 years, compared with individuals with normal weight at both 8 and 20 years (Figure 3B).

**Figure 4.**
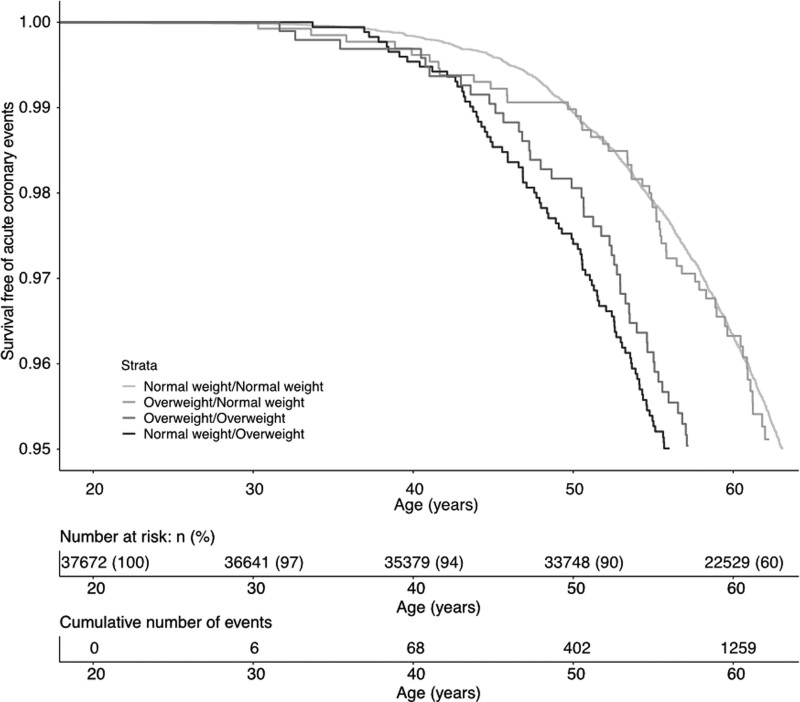
**Kaplan-Meier curve of survival free of acute coronary events according to overweight status at 8 and 20 y of age in 37 672 Swedish men in the BEST (BMI Epidemiology Study) Gothenburg cohort.** The graph shows survival free of acute coronary events according to the following groups of overweight status: normal weight/normal weight=not overweight at 8 or 20 y of age, overweight/normal weight=overweight at 8 but not at 20 y of age, normal weight/overweight=overweight at 20 but not at 8 y of age, overweight/overweight=overweight both at 8 and 20 y of age. Childhood overweight at 8 y of age^24^ was defined as BMI ≥17.9 kg/m^2^ while young adult overweight at 20 y of age was defined as BMI ≥25 kg/m^2^. The *P* values for comparison between the groups assessed by log-rank and adjusted for multiple testing (3 comparisons) were <0.001 for normal weight/overweight and overweight/overweight vs normal weight/normal weight, and not significant for overweight/normal weight vs normal weight/normal weight.

In addition, we observed unaltered results in models adjusted for birthweight (Table III in the Data Supplement) or education level (Table IV in the Data Supplement) as well as in a subgroup only including men born in Sweden and whose both parents were born in Sweden (Table V in the Data Supplement). A Kaplan-Meier survival plot analysis confirmed a higher risk of acute coronary events for men who became overweight during puberty and those who were overweight throughout childhood and puberty, but not those overweight at 8 years who normalized their weight during puberty, compared with those who were never overweight (Figure 4). Cumulative incidence plots of acute coronary events and nonacute coronary events mortality did not indicate that there was competing nonacute coronary mortality influencing the findings (Figure IA and IB in the Data Supplement).

### Association Between Childhood and Young Adult Overweight and Risk of Death Due to the Acute Coronary Event, Among Subjects With a First Acute Coronary Event in the BEST Gothenburg Cohort

To investigate the role of developmental overweight status for the severity of the acute coronary event, we also evaluated the association between the overweight groups and the risk of death due to the acute coronary events among individuals with a first adult acute coronary event (Figure 3C). Individuals who became overweight during puberty had an increased risk of death due to the acute coronary event, compared with men who were never overweight (hazard ratio, 1.51 [1.09–2.08], Figure 3C). In contrast, neither the group that was overweight throughout childhood and adolescence, nor the group with overweight at 8 years of age that normalized during puberty, had an increased risk of death due to the acute coronary event compared with men who were never overweight (Figure 3C). Thus, among individuals with acute coronary events in adulthood, specifically overweight with pubertal onset is associated with increased risk of death in connection with the coronary event.

### The Direct Association Between Pubertal BMI Change and the Risk of Acute Coronary Events in the BEST Gothenburg Cohort Is Nonlinear

When evaluated as a continuous parameter, pubertal BMI change displayed a robust, nonlinear (*P*<0.001 for nonlinearity) association with the risk of acute coronary events. Due to violation of the assumption of proportional hazards for pubertal BMI change as a continuous parameter, the follow-up was split into early and late acute coronary events. Individuals in the upper quartile of pubertal BMI change was associated with substantially increased risk of both early and late acute coronary events, compared with the individuals in the other 3 quartiles, independently of childhood BMI (Table VI in the Data Supplement). The association between pubertal BMI change and the risk of adult acute coronary events was further evaluated using a restricted cubic spline approach, confirming a statistically significant nonlinear association for both early (Figure IIA in the Data Supplement) and late (Figure IIB in the Data Supplement) acute coronary events with the most pronounced direct association observed for the men in the upper quartile of pubertal BMI change. When adjusted for pubertal BMI change, childhood BMI evaluated as a continuous parameter displayed a modest linear association with early but not late risk of acute coronary events (Table III in the Data Supplement).

## Discussion

There is a knowledge gap regarding the association between pubertal BMI change and adult coronary atherosclerosis as analyzed by computed tomography imaging and the risk of adult acute coronary events. In our analysis, where we linked data on childhood BMI and pubertal BMI change to midlife CAC score, an assessment of coronary atherosclerosis, we found that pubertal BMI change was directly associated with CAC score, independently of midlife BMI and major cardiovascular risk factors. In addition, linking the BEST Gothenburg cohort of 37 672 individuals to national registers, we observed that subjects who developed overweight during puberty had increased risk of a first acute coronary event and of dying in association with the event.

The association between BMI during development and coronary artery atherosclerosis is not well studied. In the Bogalusa Heart Study, atherosclerosis in the coronary arteries, assessed as the presence of fatty streaks and fibrous plaques at autopsy, was evaluated in a small sub-cohort of 93 individuals at age 2 to 39 years.^19^ The authors report a significant correlation between BMI and the presence of fatty streaks and fibrous plaques. Whether or not there was a correlation specifically with BMI during developmental ages such as childhood or puberty was not reported. In the Avon Longitudinal Study of Parents and Children cohort, individuals with high fat mass between 9 and 17 years of age had higher arterial stiffness, an early marker of atherosclerosis, in the carotid and femoral arteries at age 17.^20^ However, the association between pubertal BMI change and markers of adult atherosclerosis in the coronary arteries has not been evaluated. Using the BEST Gothenburg cohort, with BMI available during both childhood and puberty, linked to the SCAPIS cohort with computed tomography analysis of the heart, we had the unique opportunity to evaluate the association between pubertal BMI change and calcifications in the coronary arteries. We, herein, demonstrate that pubertal BMI change, but not childhood BMI at age 8, was directly associated with midlife CAC score. Importantly, pubertal BMI change was directly associated with CAC score, independent of midlife BMI, demonstrating that a large pubertal BMI change contributes information beyond that of tracking into a high adult BMI. Two recent Mendelian randomization studies demonstrated that the associations between BMI in childhood and adult CHD and asthma were mainly mediated through a high BMI in midlife.^7,8^ No studies have so far evaluated the associations for pubertal BMI change using the Mendelian randomization approach, but in the present observation study, pubertal BMI change was associated with CAC score independent of midlife BMI. The observation that pubertal BMI change only explained 14% of the variation in midlife BMI supports the notion that pubertal BMI change may be an independent risk marker with unique information, not captured by midlife BMI. Furthermore, the finding that the strength of the association between pubertal BMI change and CAC score was not attenuated when the cardiovascular risk factors were included in the model, indicates that it is unlikely that the observed association is mediated to a substantial part via any of these 3 risk factors and demonstrate that a high pubertal BMI increase is a novel independent risk marker of atherosclerosis. These findings support the idea that the atherosclerotic process starts already during puberty, and that the pubertal period may constitute an important window for prevention.

Some previous studies have evaluated the association between one BMI measurement during development or young adulthood and adult CHD. In a well-powered study on Israeli army personnel, young adult BMI at 17 years of age was a significant predictor of adult CHD.^11^ However, since only one BMI measurement (after puberty) was available in that study it was not possible to determine the relative importance of childhood BMI and pubertal BMI change. Thus, while both young adult BMI^9–11^ and BMI during development^5,6,21,22^ have been shown to associate separately with increased risk of CHD, the lack of well-powered studies with BMI measurements available both before and directly after puberty has made it difficult to determine the independent role of pubertal BMI change for the risk of adult acute coronary events. Childhood and puberty represent 2 physiologically distinct developmental periods, and this notion is supported by our observations that childhood BMI and pubertal BMI change only correlate marginally and that pubertal BMI change is, independently of childhood BMI, a determinant of acute coronary events in the present study, and as previously reported, of cardiovascular mortality,^12^ stroke,^13^ heart failure,^14^ and asthma.^15^ In the present study, we demonstrate that individuals with pubertal onset overweight have higher risk of acute coronary events, and of death due to the acute coronary events, compared with individuals that were never overweight. In contrast, individuals with childhood overweight with remission during puberty did not have an increased risk of acute coronary events compared with those that were never overweight. However, a direct association was seen for childhood BMI at 8 years of age and the risk of early acute coronary events, in line with previous findings.^5,6,21^ In addition, we made the important finding that among individuals who suffered an acute coronary event, specifically those with pubertal onset overweight had an increased risk of death due to the acute coronary event, compared with the never overweight. These findings demonstrate that excessive increase of BMI during puberty increases the risk and may increase the severity of adult acute coronary events.

The finding that overweight during the childhood years that is reversed before the age of 20 is not associated with a persistent increase in the risk for acute coronary events indicates that interventions leading to a healthier BMI before reaching adult age may positively alter the life course risk of cardiovascular disease for individuals with overweight in childhood.

The potential mechanisms behind the association between the pubertal BMI change and CAC score and acute coronary events are not known. The observational design of the present study precludes causal conclusions based on the observed associations, but our findings could be useful for hypothesis generation. We have previously demonstrated that a large pubertal BMI change is associated with more visceral adipose tissue in young adult age.^23^ One may, therefore, speculate that pubertal expansion of the visceral fat depot might at least partly mediate the observed associations in the present study. Based on our findings, we propose that the pubertal period might be an important window to identify boys with excessive BMI increase during puberty for targeted primary prevention to avoid adult coronary artery disease. The limitations of the present study include that the results may have limited generalizability to other ethnicities. As Sweden did not have mandatory female military conscription, we were unable to retrieve young adult BMI for women, which made it impossible for us to determine the associations between the developmental BMI parameters and risk of acute coronary events in women. Furthermore, the lack of confounding control is an important limitation. Data on cardiovascular risk factors and confounders such as demographics, family income, diet, and physical activity during included individuals’ childhood were not available and hence, the analyses could not be adjusted for these possible confounders. The strengths of the study include that we were able to link developmental BMI data to information on CAC score, a marker of coronary atherosclerosis, midlife BMI, and detailed information on cardiovascular risk factors, and to information on coronary artery events from high-quality national registers for individuals in the same population.

In conclusion, pubertal BMI change is an independent predictor of CAC score and risk of acute coronary events in adult men. These findings suggest that excessive BMI increase during puberty initiates the coronary atherosclerotic process, and thereby increases the risk and severity of adult acute coronary events.

## Sources of Funding

This study was supported by the Swedish Research Council (Dr Kindblom: 2018-02597 and Dr Ohlsson: 2016-01001) the Heart-Lung Foundation (Dr Kindblom: 20190624; 20190404)), and by grants from the Swedish state under the agreement between the Swedish government and the county councils, the ALF-agreement (Dr Kindblom: ALFGBG-723791, Dr Ohlsson: ALFGBG-720331), the Lundberg Foundation (Dr Ohlsson: 2017-0081), the Torsten Söderberg Foundation (Dr Ohlsson: M65/15), the Novo Nordisk Foundation (Dr Ohlsson: NNF180C0033898), the Knut and Alice Wallenberg Foundation (Dr Ohlsson: KAW2015.0317). The main funding body of the SCAPIS (Swedish Cardiopulmonary Bioimage Study) is the Swedish Heart-Lung Foundation. The study is also funded by the Knut and Alice Wallenberg Foundation, the Swedish Research Council and VINNOVA (Sweden’s innovation agency), the University of Gothenburg and Sahlgrenska University Hospital, Karolinska Institutet and Stockholm county council, Linköping University and University Hospital, Lund University and Skåne University Hospital, Umeå University and University Hospital, Uppsala University and University Hospital. The funding sources have not been involved in study design, data collection, analysis, and interpretation of data or in writing the article or in the decision to submit the article for publication. There was no commercial sponsorship.

## Disclosures

None.

## Supplemental Materials

Expanded Methods

Expanded Results

Online Tables I–VI

Online Figures I–IV

Online Legends to Figure

Online Figures SI–SII

References ^25–28^

## Supplementary Material


