# Supplementary figures and images for: Pubertal Body Mass Index Change Is Associated With Adult Coronary Atherosclerosis and Acute Coronary Events in Men
Source: Arterioscler Thromb Vasc Biol. 2021 Jun 17;41(8):2318–27. doi: 10.1161/ATVBAHA.121.316265 (PMC8288483; doi:10.1161/ATVBAHA.121.316265)

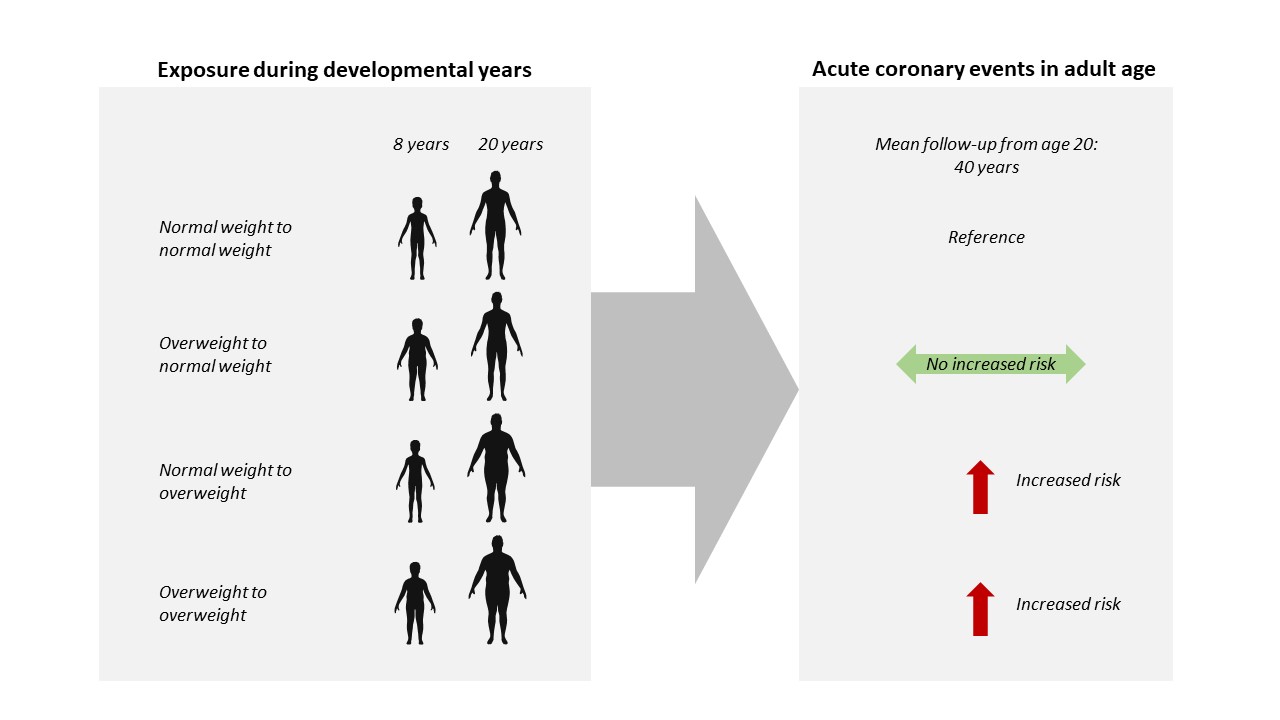

Supplement: Supplementary file 1 [file atv-41-2318-s001.jpg]
