# Supplementary material for: Pubertal Body Mass Index Change Is Associated With Adult Coronary Atherosclerosis and Acute Coronary Events in Men
Source: Arterioscler Thromb Vasc Biol. 2021 Jun 17;41(8):2318–27. doi: 10.1161/ATVBAHA.121.316265 (PMC8288483; doi:10.1161/ATVBAHA.121.316265)
Supplement: Supplementary file 2 [file atv-41-2318-s002.pdf]

SUPPLEMENTAL MATERIALS

Pubertal BMI Change is Associated Adult Coronary Atherosclerosis and Acute Coronary Events in Men

Jenny M Kindblom*, Maria Bygdell*, Ola Hjelmgren, Jari Martikainen, Annika Rosengren, Göran Bergström, Claes Ohlsson

**contributed equally*

Affiliations:

[1] Department of Internal Medicine and Clinical Nutrition, Institute of Medicine, Centre for Bone and Arthritis Research, the Sahlgrenska Academy at University of Gothenburg

[2] Region Västra Götaland, Sahlgrenska University Hospital, Pediatric Clinical Research Center, Gothenburg, Sweden

[3] Department of Molecular and Clinical Medicine, Institute of Medicine, Sahlgrenska Academy, University of Gothenburg, Gothenburg, Sweden

[4] Region Västra Götaland, Sahlgrenska University Hospital, Department of Clinical Physiology, Gothenburg, Sweden

[5]Bioinformatics Core Facility, the Sahlgrenska Academy at the University of Gothenburg, Gothenburg, Sweden

[6] Region Västra Götaland, Sahlgrenska University Hospital, Department of Medicine/Östra, Gothenburg, Sweden

[7] Region Västra Götaland, Sahlgrenska University Hospital, Department of Drug Treatment, Gothenburg, Sweden

Corresponding author: Jenny M Kindblom, Klinfarmlab, Vita stråket 11, Sahlgrenska University Hospital, S-413 45 Gothenburg, Sweden, jenny.kindblom@gu.se +46 709 902201

Supplementary Material

Methods

The BMI Epidemiology Study Gothenburg

The BMI Epidemiology Study Gothenburg (BEST Gothenburg) was initiated with the overall aim to study the impact of BMI during childhood and puberty on adult diseases. To that end we collected data on birthweight as well as directly measured height and weight throughout childhood from centrally archived School Health Care (SHC) records for all men born 1945 to 1961 in Gothenburg, Sweden. We also collected height and weight at young adult age from military conscription tests, kept by the Swedish Defense Recruitment Agency. Conscription was mandatory until 2010 for all Swedish men. The study cohort was linked to national disease registers using the Personal Identity Numbers (PIN) from the included participants. Eligible individuals were those with a SHC record in the central archive and a ten-digit PIN. Participants with data available for calculation of both childhood and young adult BMI were included in the present study (n=37,672).

The SCAPIS study

The Swedish CArdio Pulmonary bioImage Study (SCAPIS) cohort includes a randomly selected population sample representing Swedish adults aged 50 to 64 years, recruited from the census register (around 50% participation rate). The study participants underwent a thorough cardiovascular characterization during two days, including extensive imaging and functional studies of the heart, lungs, and metabolism. They also completed an extensive questionnaire regarding lifestyle and living conditions[16]. The SCAPIS study has been approved by the Ethical Review Board of Umeå, Sweden.

Exposures

Pre-pubertal childhood BMI at 8 years of age and young adult BMI at 20 years of age were calculated using all paired height and weight measurements in the period between 6.5 and 9.5 years of age for pre-pubertal childhood BMI, and in the period 17.5 to 22 years of age for young adult BMI. All paired measurements within these intervals were used to construct a linear regression model and the data for individual subjects were then corrected on this regression to obtain BMI at 8 and 20 years of age. After this correction, the BMIs were used

to classify subjects as overweight and obese at 8 years of age (using the Centers for Disease Control and Prevention's cutoffs[24] at 8 years of age for overweight: BMI≥17.9 kg/m^2, and for obesity: BMI≥20.0 kg/m^2) and at 20 years of age (based on BMI ≥25 or 30 kg/m^2, respectively). Overweight refers to the population with a BMI above the overweight cutoff and includes both overweight and obese subjects at either 8 or 20 years of age, respectively. Pubertal BMI change was defined as the difference between young adult BMI and childhood BMI, and young adult BMI thereby is the sum of childhood BMI and pubertal BMI change.

Measurement of Coronary Artery Calcification

Coronary artery calcification (CAC) was assessed using a state-of-the-art multi-slice computed tomography scanner (Siemens, Somatom Definition Flash, Siemens Medical Solution, Forchheim, Germany). Imaging and analyses were performed using a calcium scoring protocol according to the standardization suggested by McCollough et al. and Agatston et al.[17, 25] and as previously described [26].

Linkage to national registers

Linkage to registers held by the National Board of Health and Welfare and Statistics Sweden was performed with the participants' ten-digit PIN. Dates and diagnoses for the first appearance of a diagnosis of Myocardial Infarction (MI) as main diagnosis from the National Patient Register or Coronary Heart Disease (CHD) as underlying cause of death from the Cause of Death Register were retrieved (Table SII). The MI diagnosis has been shown to have a high validity in the National Patient Register[27]. Diagnoses were coded according to the International Classification of Diseases system.

We retrieved the study subjects′ highest achieved education level during the years 1990-2012, categorized as low (elementary school), medium (secondary school) or high (university) from the Longitudinal integration database for health insurance and labor market studies held by Statistics Sweden.

Representativeness of BEST-SCAPIS sub-cohort with BEST Gothenburg

In order to investigate if those included in the BEST-SCAPIS sub-cohort are different from those included in the complete BEST Gothenburg cohort, we evaluated childhood and young adult BMI. Neither young adult BMI nor childhood BMI differed significantly between the two groups (Table 1), demonstrating that the BEST Gothenburg-SCAPIS sub-cohort is

representative of the complete BEST Gothenburg cohort. Moreover, we also compared height, weight and BMI from conscription for individuals included in the present study, with individuals not included in the present study who had a measurement from conscription available. For BMI, no significant difference was seen between the included and non-included individuals, and for height and weight, marginal differences (<0.5%) were seen (Table SI).

Statistical Analyses

Birthweight, childhood BMI, and young adult BMI were log-transformed when used in the Cox regression models. We evaluated a potential non-linear association by inclusion of a quadratic term and if significant, further evaluations were undertaken using a restricted cubic spline-approach in the Cox regression analysis for a flexible non-linear assessment of the hazard ratio (HR) in relation to pubertal BMI change[28]. Four knots placed at the pubertal BMI change percentiles 5, 50, 55 and 95 (Figure SIIA) and 5, 50, 90 and 95 (Figure SIIB), respectively, were found to give a small Akaike Information Criterion and capture the average curve shape over a systematic assessment of different alternatives. A possible interaction was assessed by addition of an interaction term (childhood BMI multiplied by pubertal BMI change) in the linear Cox regression models.

Kaplan-Meier survival plots and the test for proportionality were done in R (version 3.6.1) with the survival and rms packages. For all other statistical analyses, we used SPSS version 24 (SPSS Inc., Chicago, IL).

Results

Adjustment for birthweight, country of birth, and education level

Adjustment for birthweight (n=35,662) did not alter the observed results for the overweight groups compared with men who were never overweight, and the risk of adult acute coronary events (Table SIII). We categorized education level in three categories (low (elementary school), medium, or high (university level)) and used it as a proxy for socioeconomic status. Adjustment for the individuals´ highest achieved education level did not alter the association between childhood and young adult overweight and acute coronary events (Table SIV). The association between overweight groups and the risk of adult acute coronary events was also evaluated for the subgroup with Sweden as country of birth for the subject and both parents (n=31,407). These analyses showed similar results as in the entire cohort (Table SV).

Table SI

	**Included in study mean (SD)**	**Not included in study mean (SD)**	**Difference (%)**	**P-value**
Height at conscription (cm)	179.1 (6.4)	178.7 (6.5)	0.25	<0.001
Weight at conscription (kg)	67.9 (9.4)	67.6 (9.5)	0.44	<0.05
BMI at conscription (kg/m^2)	21.1 (2.5)	21.1 (2.6)	<0.1	NS

BMI, height and weight from conscription for individuals included in the present study compared with those not included with measurements from conscription available. Values are presented as mean (SD) and statistical significance was tested using *student´s t*-test.
Included individuals n=34,703; non-included individuals n= 4,785.
NS= not significant.

Table SII Definition of the outcomes acute coronary events and fatal acute coronary events according to the International Classification of Diseases system

Event	ICD10	ICD9	ICD8
Acute Coronary event	I21[#]<br>I20-I25[*]	410[#]<br>410-414[*]	410[#]<br>410-414[*]
Fatal Acute Coronary event	I20-I25[*]	410-414[*]	410-414[*]

Definition of outcomes according to the International Classification of Diseases system. Death due to a fatal coronary event 28 days or less after an initial acute coronary event was assessed as related and therefore regarded as one event.

[#]From the National Patient Register as main diagnosis. [*]From the Cause of Death Register as underlying cause of death.

Table SIII Acute coronary events according to overweight at 8 years of age (childhood) and/or at 20 years of age (young adult age) after adjustment for birthweight

Childhood/Young adult BMI status	Events	HR (95% CI)
Normal weight/Normal weight (n=31,714)	1,473	1 (reference)
Overweight/Normal weight (n=1,292)	67	1.17 (0.91; 1.49)
Normal weight/Overweight (n=1,714)	144	2.06 (1.74; 2.45)
Overweight/Overweight (n=942)	70	1.73 (1.36; 2.20)

Hazard Ratios (HRs) for the risk of an acute coronary event were calculated using Cox proportional hazards regression in a sub-group of men with birthweight available (n=35,662). Normal weight/Normal weight = Not overweight at 8 or 20 years of age, Overweight/Normal weight = Overweight at 8 but not at 20 years of age, Normal weight/Overweight = Overweight at 20 but not at 8 years of age, Overweight/Overweight = Overweight both at 8 and 20 years of age. Childhood overweight at 8 years of age was defined as BMI ≥17.9 kg/m^2 [24] while young adult overweight at 20 years of age was defined as BMI ≥25 kg/m^2. The model has been adjusted for birth year, country of birth, and birthweight.
CI= Confidence Interval.

Table SIV Acute coronary events according to overweight at 8 years of age (childhood) and/or at 20 years of age (young adult age) adjusted for maximal education level

Childhood/Young adult BMI status	Events	HR (95% CI)
Normal weight/Normal weight (n=32,523)	1,554	1 (reference)
Overweight/Normal weight (n=1,334)	70	1.19 (0.94; 1.52)
Normal weight/Overweight (n=1,754	154	2.01 (1.71; 2.38)
Overweight/Overweight (n=961)	73	1.71 (1.35; 2.16)

Hazard Ratios (HRs) for the risk of an acute coronary event were calculated using Cox proportional hazards regression adjusted for maximal education level available for a sub-group (n=36,572). Normal weight/Normal weight = Not overweight at 8 or 20 years of age, Overweight/Normal weight = Overweight at 8 but not at 20 years of age, Normal weight/Overweight = Overweight at 20 but not at 8 years of age, Overweight/Overweight = Overweight both at 8 and 20 years of age. Childhood overweight at 8 years of age was defined as BMI ≥17.9 kg/m^2 [24] while young adult overweight at 20 years of age was defined as BMI ≥25 kg/m^2. The model has been adjusted for birth year, country of birth, and maximal achieved education level.

CI= Confidence Interval.

Table SV Acute coronary events according to overweight at 8 years of age (childhood) and/or at 20 years of age (young adult age) in men born in Sweden with parents born in Sweden

Childhood/Young adult BMI status	Events	HR (95% CI)
Normal weight/Normal weight (n=27,979)	1,318	1 (reference)
Overweight/Normal weight (n=1,138)	57	1.10 (0.85; 1.44)
Normal weight/Overweight (n=1,469)	129	2.10 (1.75; 2.52)
Overweight/Overweight (n=821)	62	1.78 (1.38; 2.29)

Hazard Ratios (HRs) for the risk of an acute coronary event were calculated using Cox proportional hazards regression in a sub-group of men born in Sweden and with parents born in Sweden (n=31,407). Normal weight/Normal weight = Not overweight at 8 or 20 years of age, Overweight/Normal weight = Overweight at 8 but not at 20 years of age, Normal weight/Overweight = Overweight at 20 but not at 8 years of age, Overweight/Overweight = Overweight both at 8 and 20 years of age. Childhood overweight at 8 years of age was defined as BMI ≥17.9 kg/m^2 [24] while young adult overweight at 20 years of age was defined as BMI ≥25 kg/m^2. The model has been adjusted for birth year.
CI= Confidence Interval.

Table SVI Adjusted Hazard Ratios for acute coronary events in relation to childhood BMI and pubertal BMI change in 37,672 Swedish men followed for a mean of 39.9 (9.4) years after age 20 years

	**Separate analyses**		**Mutually adjusted analysis**	
	**Childhood BMI** HR (95% CI) per SD increase	**ΔpBMI** Q4 vs Q1-3 HR (95% CI)	**Childhood BMI** HR (95% CI) per SD increase	**ΔpBMI** Q4 vs Q1-3 HR (95% CI)
**Events**				
Early	1.13 (1.07-1.21)	1.52 (1.32; 1.74)	1.12 (1.05-1.19)	1.48 (1.29; 1.70)
Late	1.07 (1.00-1.14)	1.24 (1.07; 1.44)	1.06 (0.99-1.13)	1.22 (1.06; 1.42)

Hazard Ratios (HRs) were calculated using Cox proportional hazards regression . ΔpBMI= pubertal BMI change, CI= confidence interval, SD= standard deviation. n=37,672. In the separate analyses, childhood BMI or ΔpBMI were included together with the covariates birth year and country of birth, and in the mutually adjusted analysis both childhood BMI and ΔpBMI were included together with the covariates birth year and country of birth. All analyses were adjusted for birth year and country of birth.

Legends to Figures

Figure SI. Cumulative incidence plots for acute coronary events (A) and non-acute coronary event mortality (B). The incidence of acute coronary events and non-acute coronary event mortality according to overweight groups for childhood and young adult overweight. Normal weight/normal weight (black line; n=33,514), overweight/normal weight (red line; n=1,368), normal weight/overweight (green line; n=1800) and overweight/overweight (blue line; n=990). Childhood overweight at 8 years of age was defined as BMI ≥17.9 kg/m^2 [24] while young adult overweight at 20 years of age was defined as BMI ≥25 kg/m^2. Cumulative incidence in each group is shown as the number of events at a given time point divided by the number of included subjects in that group. Shaded area represents 95% confidence interval.

Figure SII Hazard ratios (HRs) for acute coronary events according to pubertal BMI change.

Cox regression analysis using a restricted cubic spline-approach for a flexible non-linear assessment of the hazard ratio (HR) for early (A) ad late (B) acute coronary events after 20 years of age in relation to pubertal BMI change (ΔpBMI). Four knots were placed at the ΔpBMI percentiles 5, 50, 55 and 95 ($p<0.05$ for non-linearity) (A) and 5, 50, 90, and 95 ($p<0.01$ for non-linearity) (B) and are indicated by vertical black lines. The models were adjusted for birth year and country of birth. Data is presented as hazard ratio (red line) ± the 95% confidence interval (blue line). The distribution of participants according to pubertal BMI change is shown in gray in the lower part of the figure. The horizontal dashed line corresponds to the reference HR of 1.0 (no excess rate of events).

Figures

Figure SIA. Cumulative incidence of acute coronary events

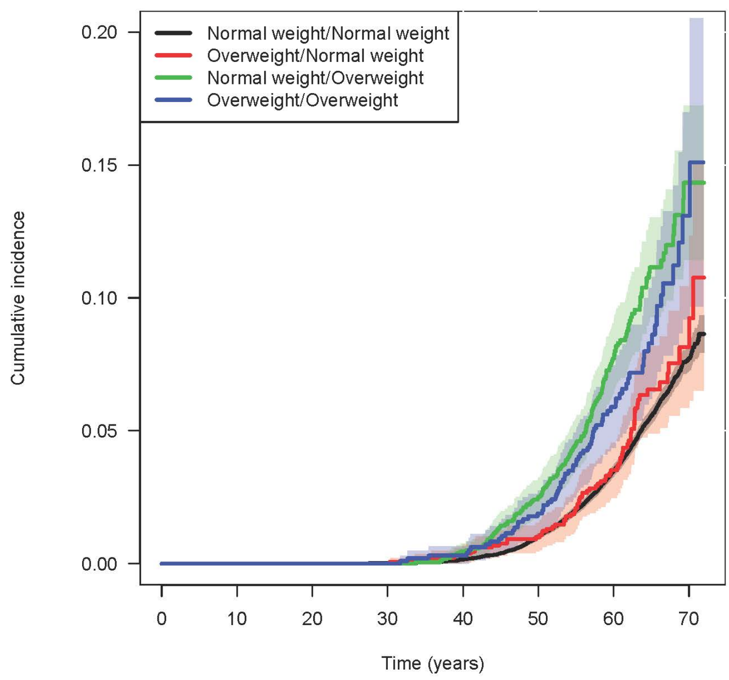

Figure SIB. Cumulative incidence of non-acute coronary event mortality

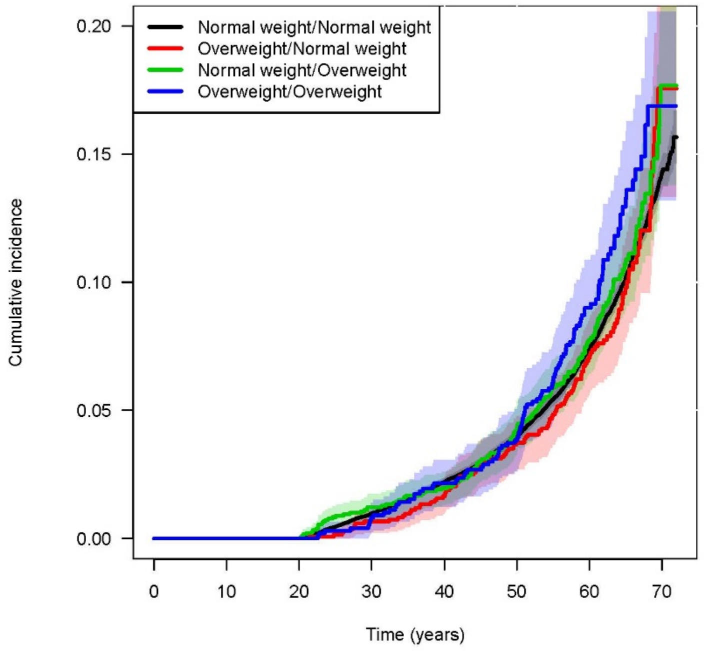

Figure SIIA. Hazard ratios (HRs) for early acute coronary events according to pubertal BMI change

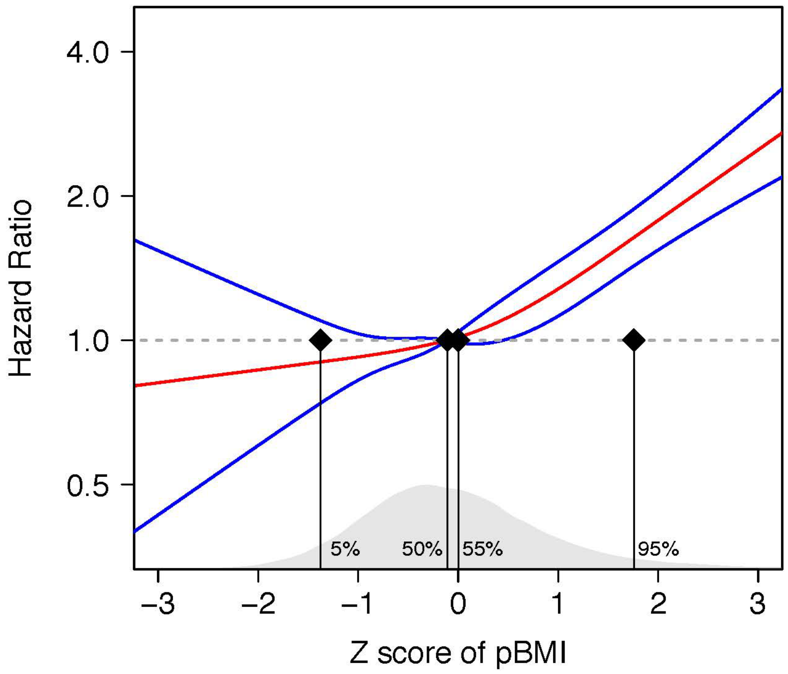

Figure SIIB. Hazard ratios (HRs) for late acute coronary events according to pubertal BMI change

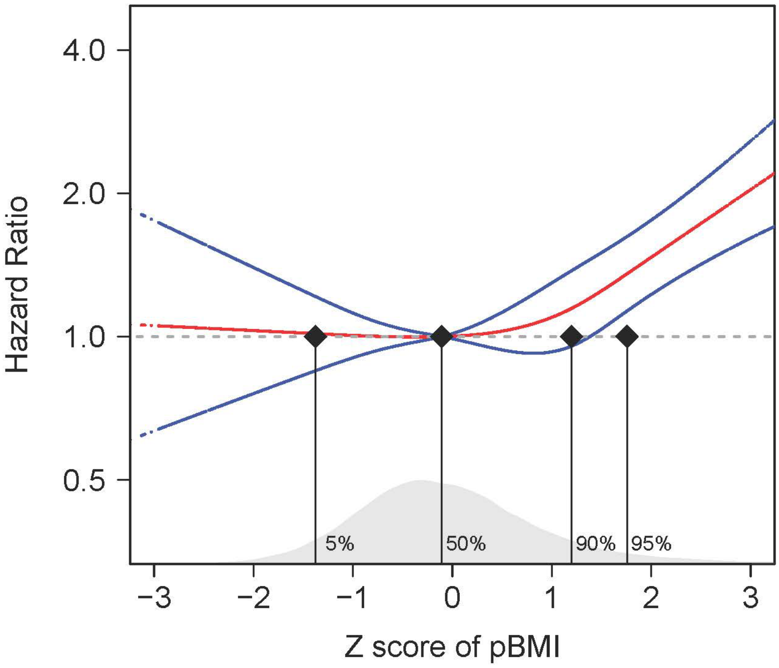
4.0
2.0
1.0
0.5
Hazard Ratio
5%
50%
90%
95%
−3
−2
−1
0
1
2
3
Z score of pBMI

Major Resources Table

In order to allow validation and replication of experiments, all essential research materials listed in the Methods should be included in the Major Resources Table below. Authors are encouraged to use public repositories for protocols, data, code, and other materials and provide persistent identifiers and/or links to repositories when available. Authors may add or delete rows as needed.

Animals (in vivo studies)

Species	Vendor or Source	Background Strain	Sex	Persistent ID / URL
NA				

Genetically Modified Animals

	Species	Vendor or Source	Background Strain	Other Information	Persistent ID / URL
**Parent - Male**	NA				
**Parent - Female**	NA				

Antibodies

Target antigen	Vendor or Source	Catalog #	Working concentration	Lot # (preferred but not required)	Persistent ID / URL
NA					

DNA/cDNA Clones

Clone Name	Sequence	Source / Repository	Persistent ID / URL
NA			

Cultured Cells

Name	Vendor or Source	Sex (F, M, or unknown)	Persistent ID / URL
NA			

Data & Code Availability

Description	Source / Repository	Persistent ID / URL
The data that support the findings of this study are available from the corresponding author upon reasonable request and upon approval from the University of Gothenburg according to mandatory national law, but are not publicly available due to privacy and ethical restrictions.		

Other

Description	Source / Repository	Persistent ID / URL
NA		

DOI [to be added]
